# Successful Treatment of Severe Type B Lactic Acidosis in a Patient with HIV/AIDS-Associated High-Grade NHL

**DOI:** 10.1155/2018/9093623

**Published:** 2018-09-13

**Authors:** Marco Mejia, Ariel Perez, Harold Watson, Daniel Sanchez, Jorge Parellada, Mario Madruga, S. J. Carlan

**Affiliations:** ^1^University of Central Florida College of Medicine, Orlando Regional Healthcare, Orlando, FL 32827, USA; ^2^Department of Medicine, Orlando Regional Healthcare, Orlando, FL, USA; ^3^Department of Pathology, Orlando Regional Healthcare, Orlando, FL, USA; ^4^Division of Academic Affairs and Research, Orlando Regional Healthcare, Orlando, FL, USA

## Abstract

Type B lactic acidosis is a rare metabolic complication sometimes associated with hematologic malignancies. When present, this type of lactic acidosis is most commonly seen in patients with high-grade lymphomas or leukemias and is usually indicative of a dismal prognosis. We report a case of a 27-year man with acquired immunodeficiency syndrome (AIDS) that presented with bilateral lower extremity swelling, an abdominal mass, and weight loss. His lab values showed elevated anion gap with lactic acidosis and computed tomography (CT) of the abdomen showed a large soft-tissue mass arising from the left hepatic lobe. Biopsy of the abdominal mass demonstrated a high-grade diffuse large B-cell lymphoma. The patient's lactic acidosis resolved after starting chemotherapy, and a complete response was evident on PET-CT after a third cycle of rituximab, etoposide, prednisone, vincristine, cyclophosphamide, and doxorubicin (EPOC-RR). Care-givers should be aware of the implications of lactic acidosis associated with malignancy and the need for prompt diagnosis and treatment.

## 1. Introduction

Lactic acidosis is a life-threatening metabolic disorder caused by elevations in blood lactate that is subclassified as Type A or Type B [1, 2]. Type A lactic acidosis reflects of poor oxygen delivery to tissues caused by hypoperfusion and is the type most frequently seen in clinical practice [3]. Type B lactic acidosis occurs under physiologic conditions where tissues remain normally perfused [2]. In some rare cases, type B lactic acidosis has been associated with solid and hematologic malignancies, such as non-Hodgkin's lymphoma (NHL) [4]. In these cases, lactic acidosis has been indicative of a poor prognosis with a mortality rate that exceeds 80%. Severe extracellular acidity due to lactic acidosis can cause cellular dysfunction and lead to cardiac arrhythmias, lowered blood pressure, and suppression of the cardiovascular response to catecholamines [5]. The current management of these patients is mainly directed at treating the underlying disease, and in cases associated with malignancy, outcomes have remained poor despite treatment with systemic therapy [1, 2]. While there is extensive literature about HIV-associated NHL and its management, only a small number of cases presenting with severe type B lactic acidosis have been reported [6].

## 2. Case Presentation

A 24-year-old male with a past medical history of HIV not on antiretroviral treatment presented to the emergency department of a large community hospital with complaints of bilateral lower extremity swelling of two weeks duration and worsening abdominal discomfort. He reported a 6-month history of mild to moderate constant abdominal pain, dull in nature, associated with distention and early satiety. He denied pain in the lower extremities. A review of systems was positive for night sweats, intermittent dry cough, shortness of breath on exertion, fatigue, poor appetite, and a 30-pound weight loss. The patient admitted to drinking alcoholic beverages on social occasions but denied tobacco or illicit drug use. He had previously been diagnosed with HIV at another hospital three years prior, but he had not been taking the antiretroviral treatment (ART) for two years.

In the emergency department, the patient's vital signs were normal and there were no signs of hypotension, sepsis, or hypoxia. On physical examination he appeared cachectic and had oral candidiasis and a large protuberant abdomen. On abdominal examination there was a large, firm, nontender mass palpated extending from the epigastrium to the periumbilical region, as well as hepatomegaly and right upper quadrant tenderness. Marked bilateral lower extremity edema up to the knee level was also noted.

Initial laboratory tests demonstrated an anion gap metabolic acidosis (anion gap 17 and HCO3 23 meq/L). Venous lactate and lactate dehydrogenase levels were both markedly elevated at 9.4 mmol /L (normal range = 0.5-1.0 mmol/L) and 2445 U/L, respectively. Aspartate aminotransferase (AST) was elevated at 63 U/L, but alanine aminotransferase (ALT) and alkaline phosphatase (ALP) were within normal limits. Uric acid was measured as 9.6 mg/dL. The remainder of the metabolic panel, including creatinine and glomerular filtration rate (GFR), was normal. The patient's renal function remained stable throughout the course of his hospital admission. Complete blood count demonstrated a normocytic anemia (white blood cells 4.2 cells/*μ*L, hemoglobin 8.1 g/dL, platelet count 176 platelets/*μ*L, and mean corpuscular volume 85 fL), likely due to chronic disease. Lab results also showed a CD4 count of 38 cells/*μ*L (3.0%), a CD8 count of 1023 cells/*μ*L (84.0%), and an HIV viral load of 354,120 copies/mL, consistent with acquired immunodeficiency syndrome (AIDS). Infectious disease was consulted for recommendations, and the patient was started on ART (dolutegravir, tenofovir, and emtricitabine) on hospital day 3.

**Figure 1:**
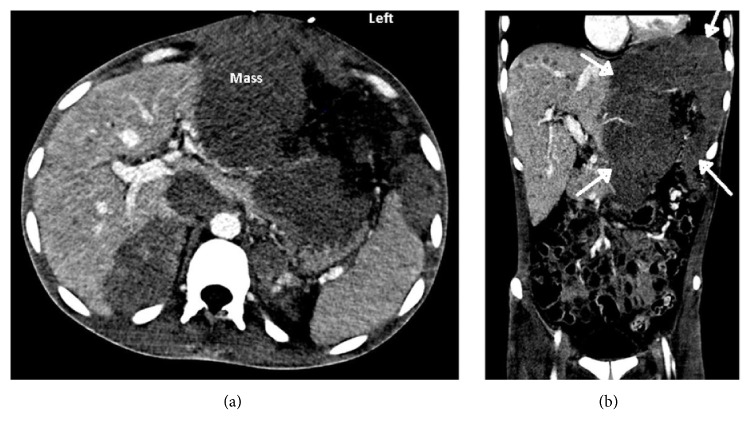
(a) Computed tomography with contrast of abdomen showing 16.3 cm × 14.1 cm × 20 cm hepatic mass (axial view). (b) Computed tomography with contrast of abdomen showing mass (arrows) involving the left hepatic lobe (coronal view).

Computed tomography (CT) with contrast of the abdomen revealed a large soft-tissue mass (16.3 cm × 14.1 cm × 20 cm) with internal vascularity that appeared to arise from the left hepatic lobe (Figure 1). Additional findings included innumerable small hypodense lesions throughout the liver parenchyma and mass effect on the inferior vena cava (IVC) by a 3.6 cm × 2.9 cm aortocaval lymph node. Additionally, there appeared to be bilateral suprarenal soft-tissue masses and sclerotic lesions within the pelvis and lower thoracic spine, suggestive of metastatic disease with bone involvement. A chest radiograph demonstrated left hilar adenopathy, a small left pleural effusion, and a left lower lobe nodular opacity. Ultrasound-guided core biopsy of the hepatic mass was performed and revealed a high-grade diffuse large B-cell lymphoma (DLBCL) (CD19+/CD20+/ CD34-/KAPPA+) with germinal center formation (FIGURE). Immunohistochemistry was positive for C-MYC, and ki-67 reactivity was greater than 90%. A bone marrow biopsy demonstrated severe hypocellularity (<5% cellularity) with atypical lymphoid infiltrate.

On hospital day 5, the patient was started on his first cycle of chemotherapy consisting of etoposide, prednisone, vincristine, cyclophosphamide, doxorubicin, and a double dose of rituximab (EPOCH-RR). Given that bone marrow involvement was present, he also received intrathecal methotrexate. By hospital day #8 the lactic acidosis resolved and was measured as 1.0 mmol/L (normal value = 0.5-1.0 mmol/L). He subsequently developed neutropenia and neuropathic pain of the hands and feet within several days of initiating chemotherapy and was treated with G-CSF until the neutropenia resolved. The patient was discharged on hospital day #26 but was readmitted the following day after an echocardiogram showed a left ventricular ejection fraction (LVEF) of 20% (normal range = 55-70%) which had decreased from 49% obtained 3 weeks earlier. He was started on Carvedilol and Lisinopril for Doxorubicin induced cardiomyopathy.

The patient completed cycles 2 and 3 of EPOC-RR without doxorubicin (due to induced cardiomyopathy) and doses of cyclophosphamide and etoposide were increased by 20% with no complications. Repeat echocardiogram also showed improvement in LVEF to 45-49% and he was discharged in stable condition. Outpatient PET-CT after the third cycle of chemotherapy showed a complete radiologic response with no remaining FDG-avid lesions. Given the complete radiologic response, the patient completed one additional cycle of EPOC-RR (fourth cycle) and was advised to follow-up with medical oncology in 3 months.

## 3. Discussion

Type B lactic acidosis is a rare complication of solid and hematologic malignancies, with only a few cases being reported in the literature. This occurs when lactate is produced despite normal oxygen delivery to tissues. It is most commonly associated with lymphoma, and it is indicative of a poor prognosis [2–4, 7]. Historically, the “Warburg effect” has been the most widely accepted hypothesis for energy metabolism in cancer cells [3]. In this model, cancer cells have greatly increased rates of glycolysis by upregulation of glycolytic enzymes, such as hexokinase, and produce lactate as a byproduct. This upregulation of glycolytic enzymes could explain why some patients with type B lactic acidosis present with hypoglycemia [4]. Recent bioenergetic studies also suggest an alternative model. These studies have demonstrated that, in some cases, stromal cells (fibroblasts, macrophages, endothelial cells, and lymphocytes) within a tumor have increased glycolytic activity, while cancer cells utilize the byproducts from stromal cell metabolism for energy production [1]. The cancer cells were observed to have decreased glucose uptake and increased metabolism through oxidative phosphorylation. This model of metabolic coupling between stromal and cancer cells has been termed the “reverse Warburg effect” [1]. This model can further be supported by the observation that lymph nodes previously involved by lymphoma may still have a high avidity for fluorodeoxyglucose on PET scans despite the eradication of cancerous cells [1]. Interestingly, many cases of lymphoma complicated by lactic acidosis also present with hepatic and renal involvement [6]. This finding has led some clinicians to believe that reduced hepatic and renal clearance of lactate contributes to the development of type B lactic acidosis [4, 6, 7].

The incidence of non-Hodgkin's lymphoma (NHL) has been reported to be approximately 80 times greater in HIV-infected patients than in noninfected patients [8]. In 2011, NHLs represented 5.1% of all cancers and occurred in approximately 2-3% of patients with AIDS [9]. The majority of HIV-associated NHLs are of B-cell origin, and these often present as aggressive malignancies with extra-nodal involvement at the time of diagnosis [6]. Due to their strong association with HIV, the 3 subtypes of NHL that most commonly occur in HIV-infected patients (diffuse large B-cell lymphoma, central nervous system lymphoma, and Burkitt lymphoma) have been classified as AIDS defining diseases by the Center for Disease Control and Prevention (CDC), with DLBCL being the most common type [6, 8].

Our patient initially presented with hepatomegaly, lower extremity edema, and significant weight loss. His laboratory results were remarkable for an elevated anion gap, lactate, lactate dehydrogenase, and mildly elevated liver enzymes. He was stable at the time of admission, and there was no evidence for another possible cause of lactic acidosis (hypotension, sepsis, organ ischemia, bowel perforation, or hypoxia). Another inciting factor to consider for lactic acidosis was use of nucleoside reverse transcriptase inhibitors (NRTIs). These medications are often a component of ART and are known to sometimes produce lactic acidosis as an adverse effect. However, our patient had not been taking ART medications for approximately 2 years, and his lactate was already elevated before he was restarted on ART so this was ruled out. Since there are multiple possible etiologies for lactic acidosis, it may often be difficult to identify a single cause. In our patient, we believe malignancy was the source of lactic acidosis given the prompt normalization of his lactate levels after beginning chemotherapy.

Biopsy of our patient's abdominal mass showed a diffuse large B-cell lymphoma (DLBLC). This is the most common lymphoid malignancy that occurs in adults, and it represents 35-40% of lymphomas in the western world [10]. In addition, while there have been many cases of HIV-associated NHL described in the literature, only a few cases complicated by lactic acidosis and hypoglycemia in the setting of liver infiltration by malignancy have been reported [6].

Currently, the only effective treatment of lactic acidosis is to reverse the cause. In cases where malignancy is thought to be the source, the mainstay of treatment is aggressive chemotherapy, although this has shown little benefit in multiple cases [4, 6, 11, 12].

In our patient, we chose to use rituximab, etoposide, prednisone, vincristine, cyclophosphamide, and doxorubicin (EPOCH-R) as some studies have shown that this regimen is superior to CHOP-R [13–15]. In patients with HIV-associated NHL, concomitant administration of ART should also be employed as this has been shown to improve outcomes and reduce the risk of opportunistic infections during chemotherapy [16].

Multiple studies have shown that lactic acidosis associated with malignancy is a poor prognostic factor [2, 4, 6, 11, 12]. An effective treatment that reduces mortality rates has yet to be identified, though some cases have reported administering thiamine or bicarbonate with very limited success [11, 12]. Others have even attempted hemodialysis, and in one case reported by Ruiz et al. they attempted all three treatment strategies. Their patient showed initial improvement but subsequently died [11, 12].

## 4. Conclusion

Type B lactic acidosis is an ominous complication that may be associated with certain tumors. Although rare, it most often presents in hematologic malignancies, with lymphomas being the most common. Currently, the most commonly employed treatment is aggressive chemotherapy in hopes that reduction of tumor burden will resolve the lactic acidosis. However, even with treatment the majority of cases complicated by lactic acidosis have resulted in death, with only a minority of reported cases living beyond a few months. Novel therapies for the treatment of malignancy associated lactic acidosis are needed, although the rarity of this condition may limit the possibility of future trials. Future studies should possibly aim to target the proposed models of type B lactic acidosis, including the “Warburg effect” and “reverse Warburg effect”.
